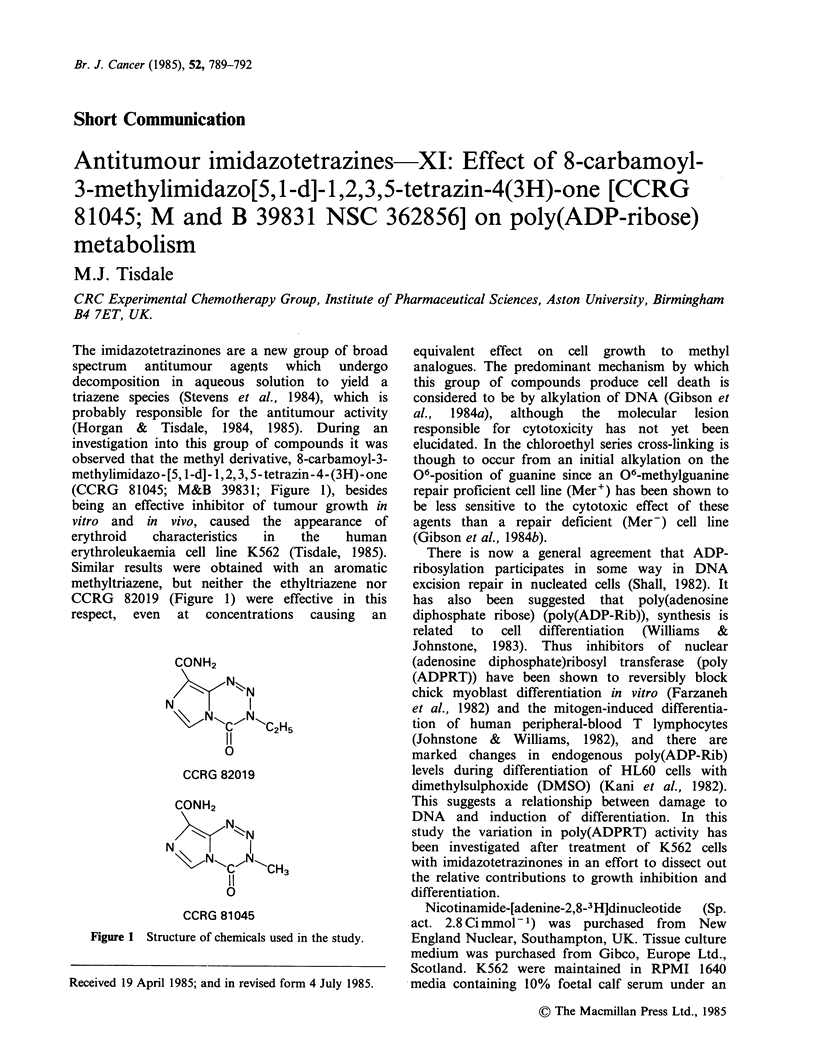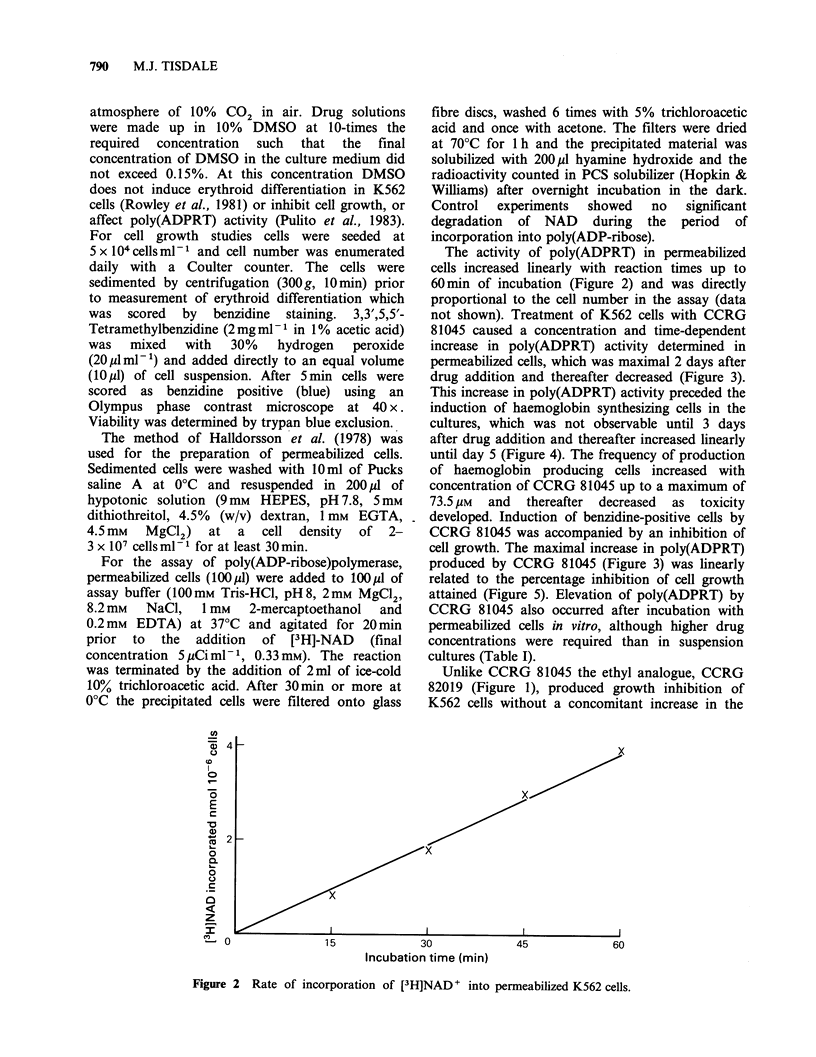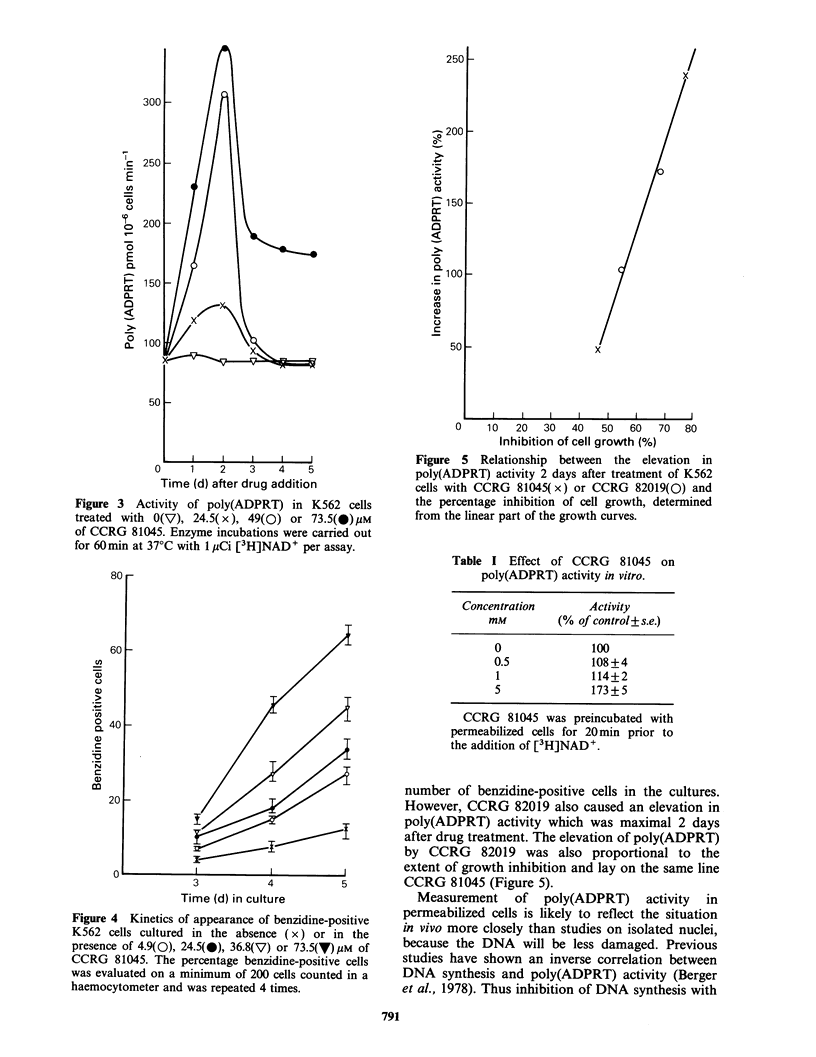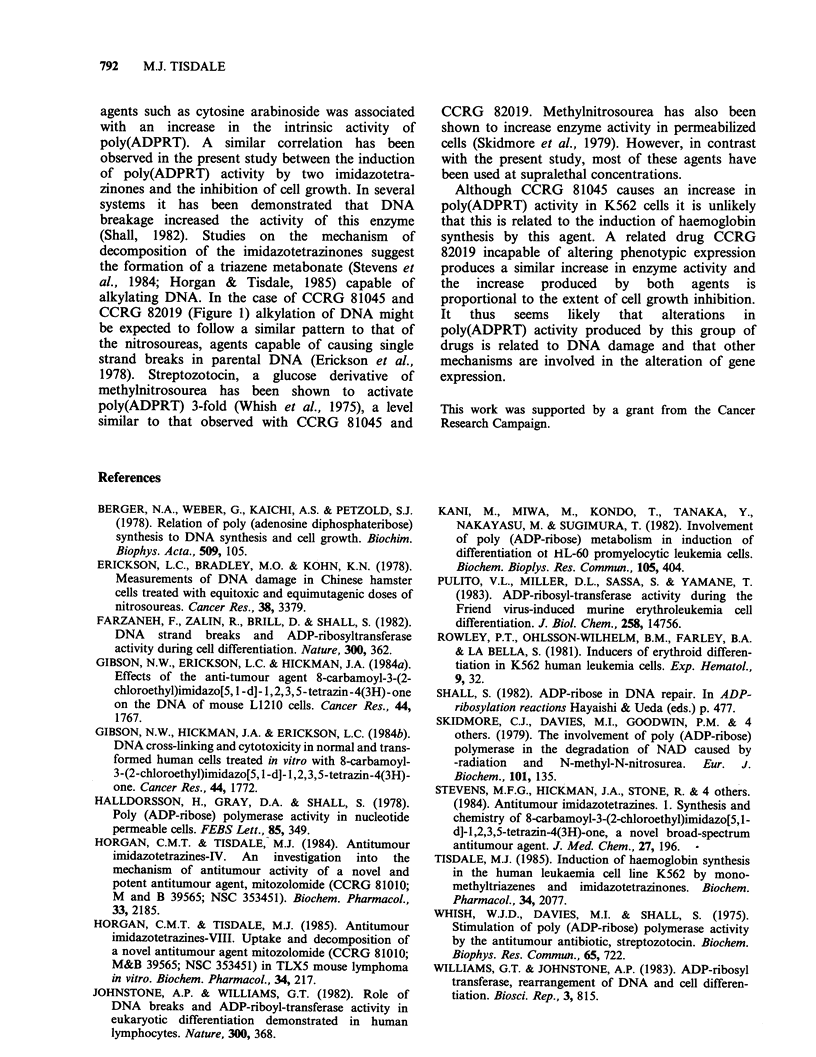# Antitumour imidazotetrazines--XI: Effect of 8-carbamoyl-3-methylimidazo[5,1-d]-1,2,3,5-tetrazin-4(3H)-one [CCRG 81045; M and B 39831 NSC 362856] on poly(ADP-ribose) metabolism.

**DOI:** 10.1038/bjc.1985.259

**Published:** 1985-11

**Authors:** M. J. Tisdale


*Br. J. Cancer* (1985), **52**, 789–792

**Short Communication**

### Antitumour imidazotetrazines—XI: Effect of 8-carbamoyl-3-methylimidazo[5,1-d]-1,2,3,5-tetrazin-4(3H)-one [CCRG 81045; M and B 39831 NSC 362856] on poly(ADP-ribose) metabolism

M.J. Tisdale

*CRC Experimental Chemotherapy Group, Institute of Pharmaceutical Sciences, Aston University, Birmingham B4 7ET, UK.*

The imidazotetrazinones are a new group of broad spectrum antitumour agents which undergo decomposition in aqueous solution to yield a triazene species (Stevens *et al.,* 1984), which is probably responsible for the antitumour activity (Horgan & Tisdale, 1984, 1985). During an investigation into this group of compounds it was observed that the methyl derivative, 8-carbamoyl-3-methylimidazo-[5,1-d]-1,2,3,5-tetrazin-4-(3H)-one (CCRG 81045; M&B 39831; Figure 1), besides being an effective inhibitor of tumour growth *in vitro* and *in vivo,* caused the appearance of erythroid characteristics in the human erythroleukaemia cell line K562 (Tisdale, 1985). Similar results were obtained with an aromatic methyltriazene, but neither the ethyltriazene nor CCRG 82019 (Figure 1) were effective in this respect, even at concentrations causing an equivalent effect on cell growth to methyl analogues. The predominant mechanism by which this group of compounds produce cell death is considered to be by alkylation of DNA (Gibson *et al.,* 1984*a*), although the molecular lesion responsible for cytotoxicity has not yet been elucidated. In the chloroethyl series cross-linking is though to occur from an initial alkylation on the $O^6$-position of guanine since an $O^6$-methylguanine repair proficient cell line ($Mer^+$) has been shown to be less sensitive to the cytotoxic effect of these agents than a repair deficient ($Mer^-$) cell line (Gibson *et al.,* 1984*b*).

CCRG 82019

CCRG 81045

**Figure 1** Structure of chemicals used in the study.

There is now a general agreement that ADP-ribosylation participates in some way in DNA excision repair in nucleated cells (Shall, 1982). It has also been suggested that poly(adenosine diphosphate ribose) (poly(ADP-Rib)), synthesis is related to cell differentiation (Williams & Johnstone, 1983). Thus inhibitors of nuclear (adenosine diphosphate)ribosyl transferase (poly (ADPRT)) have been shown to reversibly block chick myoblast differentiation *in vitro* (Farzaneh *et al.,* 1982) and the mitogen-induced differentiation of human peripheral-blood T lymphocytes (Johnstone & Williams, 1982), and there are marked changes in endogenous poly(ADP-Rib) levels during differentiation of HL60 cells with dimethylsulphoxide (DMSO) (Kani *et al.,* 1982). This suggests a relationship between damage to DNA and induction of differentiation. In this study the variation in poly(ADPRT) activity has been investigated after treatment of K562 cells with imidazotetrazinones in an effort to dissect out the relative contributions to growth inhibition and differentiation.

Nicotinamide-[adenine-2,8-$^3$H]dinucleotide (Sp. act. 2.8 Ci mmol$^{-1}$) was purchased from New England Nuclear, Southampton, UK. Tissue culture medium was purchased from Gibco, Europe Ltd., Scotland. K562 were maintained in RPMI 1640 media containing 10% foetal calf serum under an

Received 19 April 1985; and in revised form 4 July 1985.

© The Macmillan Press Ltd., 1985

atmosphere of 10% $CO_2$ in air. Drug solutions were made up in 10% DMSO at 10-times the required concentration such that the final concentration of DMSO in the culture medium did not exceed 0.15%. At this concentration DMSO does not induce erythroid differentiation in K562 cells (Rowley *et al.*, 1981) or inhibit cell growth, or affect poly(ADPRT) activity (Pulito *et al.*, 1983). For cell growth studies cells were seeded at $5 \times 10^4$ cells ml$^{-1}$ and cell number was enumerated daily with a Coulter counter. The cells were sedimented by centrifugation (300 *g*, 10 min) prior to measurement of erythroid differentiation which was scored by benzidine staining. 3,3′,5,5′-Tetramethylbenzidine (2 mg ml$^{-1}$ in 1% acetic acid) was mixed with 30% hydrogen peroxide (20 $\mu$l ml$^{-1}$) and added directly to an equal volume (10 $\mu$l) of cell suspension. After 5 min cells were scored as benzidine positive (blue) using an Olympus phase contrast microscope at 40×. Viability was determined by trypan blue exclusion.

The method of Halldorsson *et al.* (1978) was used for the preparation of permeabilized cells. Sedimented cells were washed with 10 ml of Pucks saline A at 0°C and resuspended in 200 $\mu$l of hypotonic solution (9 mM HEPES, pH 7.8, 5 mM dithiothreitol, 4.5% (w/v) dextran, 1 mM EGTA, 4.5 mM $MgCl_2$) at a cell density of 2–$3 \times 10^7$ cells ml$^{-1}$ for at least 30 min.

For the assay of poly(ADP-ribose)polymerase, permeabilized cells (100 $\mu$l) were added to 100 $\mu$l of assay buffer (100 mM Tris-HCl, pH 8, 2 mM $MgCl_2$, 8.2 mM NaCl, 1 mM 2-mercaptoethanol and 0.2 mM EDTA) at 37°C and agitated for 20 min prior to the addition of [$^3$H]-NAD (final concentration 5 $\mu$Ci ml$^{-1}$, 0.33 mM). The reaction was terminated by the addition of 2 ml of ice-cold 10% trichloroacetic acid. After 30 min or more at 0°C the precipitated cells were filtered onto glass fibre discs, washed 6 times with 5% trichloroacetic acid and once with acetone. The filters were dried at 70°C for 1 h and the precipitated material was solubilized with 200 $\mu$l hyamine hydroxide and the radioactivity counted in PCS solubilizer (Hopkin & Williams) after overnight incubation in the dark. Control experiments showed no significant degradation of NAD during the period of incorporation into poly(ADP-ribose).

The activity of poly(ADPRT) in permeabilized cells increased linearly with reaction times up to 60 min of incubation (Figure 2) and was directly proportional to the cell number in the assay (data not shown). Treatment of K562 cells with CCRG 81045 caused a concentration and time-dependent increase in poly(ADPRT) activity determined in permeabilized cells, which was maximal 2 days after drug addition and thereafter decreased (Figure 3). This increase in poly(ADPRT) activity preceded the induction of haemoglobin synthesizing cells in the cultures, which was not observable until 3 days after drug addition and thereafter increased linearly until day 5 (Figure 4). The frequency of production of haemoglobin producing cells increased with concentration of CCRG 81045 up to a maximum of 73.5 $\mu$M and thereafter decreased as toxicity developed. Induction of benzidine-positive cells by CCRG 81045 was accompanied by an inhibition of cell growth. The maximal increase in poly(ADPRT) produced by CCRG 81045 (Figure 3) was linearly related to the percentage inhibition of cell growth attained (Figure 5). Elevation of poly(ADPRT) by CCRG 81045 also occurred after incubation with permeabilized cells *in vitro*, although higher drug concentrations were required than in suspension cultures (Table I).

Unlike CCRG 81045 the ethyl analogue, CCRG 82019 (Figure 1), produced growth inhibition of K562 cells without a concomitant increase in the

**Figure 2** Rate of incorporation of [$^3$H]NAD$^+$ into permeabilized K562 cells.

**Figure 3** Activity of poly(ADPRT) in K562 cells treated with 0(▽), 24.5(×), 49(○) or 73.5(●) μM of CCRG 81045. Enzyme incubations were carried out for 60 min at 37°C with 1 μCi [$^3$H]NAD$^+$ per assay.

**Figure 4** Kinetics of appearance of benzidine-positive K562 cells cultured in the absence (×) or in the presence of 4.9(○), 24.5(●), 36.8(▽) or 73.5(▼) μM of CCRG 81045. The percentage benzidine-positive cells was evaluated on a minimum of 200 cells counted in a haemocytometer and was repeated 4 times.

**Figure 5** Relationship between the elevation in poly(ADPRT) activity 2 days after treatment of K562 cells with CCRG 81045(×) or CCRG 82019(○) and the percentage inhibition of cell growth, determined from the linear part of the growth curves.

**Table I** Effect of CCRG 81045 on poly(ADPRT) activity *in vitro*.

| *Concentration* mM | *Activity* (% *of control* ± *s.e.*) |
|---|---|
| 0 | 100 |
| 0.5 | 108 ± 4 |
| 1 | 114 ± 2 |
| 5 | 173 ± 5 |

CCRG 81045 was preincubated with permeabilized cells for 20 min prior to the addition of [$^3$H]NAD$^+$.

number of benzidine-positive cells in the cultures. However, CCRG 82019 also caused an elevation in poly(ADPRT) activity which was maximal 2 days after drug treatment. The elevation of poly(ADPRT) by CCRG 82019 was also proportional to the extent of growth inhibition and lay on the same line CCRG 81045 (Figure 5).

Measurement of poly(ADPRT) activity in permeabilized cells is likely to reflect the situation *in vivo* more closely than studies on isolated nuclei, because the DNA will be less damaged. Previous studies have shown an inverse correlation between DNA synthesis and poly(ADPRT) activity (Berger *et al.*, 1978). Thus inhibition of DNA synthesis with agents such as cytosine arabinoside was associated with an increase in the intrinsic activity of poly(ADPRT). A similar correlation has been observed in the present study between the induction of poly(ADPRT) activity by two imidazotetrazinones and the inhibition of cell growth. In several systems it has been demonstrated that DNA breakage increased the activity of this enzyme (Shall, 1982). Studies on the mechanism of decomposition of the imidazotetrazinones suggest the formation of a triazene metabonate (Stevens *et al.*, 1984; Horgan & Tisdale, 1985) capable of alkylating DNA. In the case of CCRG 81045 and CCRG 82019 (Figure 1) alkylation of DNA might be expected to follow a similar pattern to that of the nitrosoureas, agents capable of causing single strand breaks in parental DNA (Erickson *et al.*, 1978). Streptozotocin, a glucose derivative of methylnitrosourea has been shown to activate poly(ADPRT) 3-fold (Whish *et al.*, 1975), a level similar to that observed with CCRG 81045 and CCRG 82019. Methylnitrosourea has also been shown to increase enzyme activity in permeabilized cells (Skidmore *et al.*, 1979). However, in contrast with the present study, most of these agents have been used at supralethal concentrations.

Although CCRG 81045 causes an increase in poly(ADPRT) activity in K562 cells it is unlikely that this is related to the induction of haemoglobin synthesis by this agent. A related drug CCRG 82019 incapable of altering phenotypic expression produces a similar increase in enzyme activity and the increase produced by both agents is proportional to the extent of cell growth inhibition. It thus seems likely that alterations in poly(ADPRT) activity produced by this group of drugs is related to DNA damage and that other mechanisms are involved in the alteration of gene expression.

This work was supported by a grant from the Cancer Research Campaign.

### References

BERGER, N.A., WEBER, G., KAICHI, A.S. & PETZOLD, S.J. (1978). Relation of poly (adenosine diphosphateribose) synthesis to DNA synthesis and cell growth. *Biochim. Biophys. Acta.*, **509**, 105.

ERICKSON, L.C., BRADLEY, M.O. & KOHN, K.N. (1978). Measurements of DNA damage in Chinese hamster cells treated with equitoxic and equimutagenic doses of nitrosoureas. *Cancer Res.*, **38**, 3379.

FARZANEH, F., ZALIN, R., BRILL, D. & SHALL, S. (1982). DNA strand breaks and ADP-ribosyltransferase activity during cell differentiation. *Nature*, **300**, 362.

GIBSON, N.W., ERICKSON, L.C. & HICKMAN, J.A. (1984*a*). Effects of the anti-tumour agent 8-carbamoyl-3-(2-chloroethyl)imidazo[5,1-d]-1,2,3,5-tetrazin-4(3H)-one on the DNA of mouse L1210 cells. *Cancer Res.*, **44**, 1767.

GIBSON, N.W., HICKMAN, J.A. & ERICKSON, L.C. (1984*b*). DNA cross-linking and cytotoxicity in normal and transformed human cells treated *in vitro* with 8-carbamoyl-3-(2-chloroethyl)imidazo[5,1-d]-1,2,3,5-tetrazin-4(3H)-one. *Cancer Res.*, **44**, 1772.

HALLDORSSON, H., GRAY, D.A. & SHALL, S. (1978). Poly (ADP-ribose) polymerase activity in nucleotide permeable cells. *FEBS Lett.*, **85**, 349.

HORGAN, C.M.T. & TISDALE, M.J. (1984). Antitumour imidazotetrazines-IV. An investigation into the mechanism of antitumour activity of a novel and potent antitumour agent, mitozolomide (CCRG 81010; M and B 39565; NSC 353451). *Biochem. Pharmacol.*, **33**, 2185.

HORGAN, C.M.T. & TISDALE, M.J. (1985). Antitumour imidazotetrazines-VIII. Uptake and decomposition of a novel antitumour agent mitozolomide (CCRG 81010; M&B 39565; NSC 353451) in TLX5 mouse lymphoma *in vitro*. *Biochem. Pharmacol.*, **34**, 217.

JOHNSTONE, A.P. & WILLIAMS, G.T. (1982). Role of DNA breaks and ADP-riboyl-transferase activity in eukaryotic differentiation demonstrated in human lymphocytes. *Nature*, **300**, 368.

KANI, M., MIWA, M., KONDO, T., TANAKA, Y., NAKAYASU, M. & SUGIMURA, T. (1982). Involvement of poly (ADP-ribose) metabolism in induction of differentiation ot HL-60 promyelocytic leukemia cells. *Biochem. Bioplys. Res. Commun.*, **105**, 404.

PULITO, V.L., MILLER, D.L., SASSA, S. & YAMANE, T. (1983). ADP-ribosyl-transferase activity during the Friend virus-induced murine erythroleukemia cell differentiation. *J. Biol. Chem.*, **258**, 14756.

ROWLEY, P.T., OHLSSON-WILHELM, B.M., FARLEY, B.A. & LA BELLA, S. (1981). Inducers of erythroid differentiation in K562 human leukemia cells. *Exp. Hematol.*, **9**, 32.

SHALL, S. (1982). ADP-ribose in DNA repair. In *ADP-ribosylation reactions* Hayaishi & Ueda (eds.) p. 477.

SKIDMORE, C.J., DAVIES, M.I., GOODWIN, P.M. & 4 others. (1979). The involvement of poly (ADP-ribose) polymerase in the degradation of NAD caused by -radiation and N-methyl-N-nitrosurea. *Eur. J. Biochem.*, **101**, 135.

STEVENS, M.F.G., HICKMAN, J.A., STONE, R. & 4 others. (1984). Antitumour imidazotetrazines. 1. Synthesis and chemistry of 8-carbamoyl-3-(2-chloroethyl)imidazo[5,1-d]-1,2,3,5-tetrazin-4(3H)-one, a novel broad-spectrum antitumour agent. *J. Med. Chem.*, **27**, 196.

TISDALE, M.J. (1985). Induction of haemoglobin synthesis in the human leukaemia cell line K562 by monomethyltriazenes and imidazotetrazinones. *Biochem. Pharmacol.*, **34**, 2077.

WHISH, W.J.D., DAVIES, M.I. & SHALL, S. (1975). Stimulation of poly (ADP-ribose) polymerase activity by the antitumour antibiotic, streptozotocin. *Biochem. Biophys. Res. Commun.*, **65**, 722.

WILLIAMS, G.T. & JOHNSTONE, A.P. (1983). ADP-ribosyl transferase, rearrangement of DNA and cell differentiation. *Biosci. Rep.*, **3**, 815.